# The Century Bourbon

**Published:** 1870-11

**Authors:** 


### "THE CENTURY BOURBON"

Is the name of a whiskey, a demijohn of which, holding several gallons, has been sent to us to drink on trial. If we do not "take" it more largely than heretofore, the demijohn will last us one million and nineteen years; and if the Messrs. Thurber & Co., of 173 Chambers street, New York, propose waiting until we make the trial fully by using it all up, they will have to wait a long time for a "notice," unless our liquor-drinking habits undergo a remarkable change.

Educated physicians will continue to prescribe whiskey and other stimulants, and patients will follow their advice.

It is beyond dispute that bad whiskey, that adulterated liquors, are exercising a most pernicious and destructive influence over the health, and morals, and minds of vast multitudes of the people of the United States. Hence any man who will guarantee to furnish a pure, unadulterated article of stimulant, merits public patronage to the extent that such an article is really necessary and useful, and to such extent also it is a humanity to the sick and suffering.

Professor Silliman, the State Chemist of Connecticut, and connected with Yale College, the son of a learned and noble Christian father, certifies, that he has made an official analysis of a sample of the Century Bourbon, and that "it is entirely free from any deleterious substance whatever; that the absence of all traces of lead or other poisonous or hurtful thing is absolute," meaning thereby that the whiskey sent him was the product of the grain out of which it was made. We have no doubt of the truth of the analysis, of its accuracy; now if the Mess. Thurber & Co. will sell such pure product, and no other, we hope that they will receive the patronage which their integrity merits. We are not acquainted with them personally, and do not want to be considered, as the editor of a Health Journal, to advise the use of stimulants in any shape or form whatever, for whoever never tastes a drop of liquor will never die in the gutter, ruined in soul, body and estate; whoever does drink a drop, may. At the same time, we consider ourselves to be acting in the interests of a true humanity, in saying that The Century Whiskey sent us is mild in its character, pure in its quality, and unquestionably adapted to all medicinal purposes.

